# Supplementary material for: Silicon photocathode functionalized with osmium complex catalyst for selective catalytic conversion of CO2 to methane
Source: Nat Commun. 2024 Jul 13;15:5882. doi: 10.1038/s41467-024-50244-w (PMC11246507; doi:10.1038/s41467-024-50244-w)
Supplement: Supplementary file 1 — Supplementary Information [file 41467_2024_50244_MOESM1_ESM.pdf]

Supplementary Information

Silicon Photocathode Functionalized with Osmium Complex Catalyst for Selective Catalytic Conversion of CO_2 to Methane

Xing-Yi Li[1,2,ǂ], *Ze-Lin Zhu*[3,ǂ], *Fentahun Wondu Dagnaw*[1], *Jie-Rong Yu*[1], *Zhi-Xing Wu*[4], *Yi-Jing Chen*[1], *Mu-Han Zhou*[1], *Tieyu Wang*[2], *Qing-Xiao Tong*[1,5*], *Jing-Xin Jian*[1,2*]

[1]Department of Chemistry, Shantou University, Shantou, 515063, P. R. China.

[*]Emails: qxtong@stu.edu.cn; jxjian@stu.edu.cn

[2]Guangdong Provincial Key Laboratory of Marine Disaster Prediction and Prevention, Shantou University, Shantou, 515063, P. R. China.

[3]Center of Super-Diamond and Advanced Films (COSDAF) and Department of Chemistry, City University of Hong Kong, Hong Kong SAR, China.

[4]Laboratory of Organic Electronics, Department of Science and Technology (ITN), Linköping University, Norrköping, SE 60174, Sweden.

[5]Key Laboratory for Preparation and Application of Ordered Structural Material of Guangdong Province, Shantou University, Shantou, 515063, P. R. China.

[ǂ] These authors contributed equally: Xing-Yi Li, Ze-Lin Zhu.

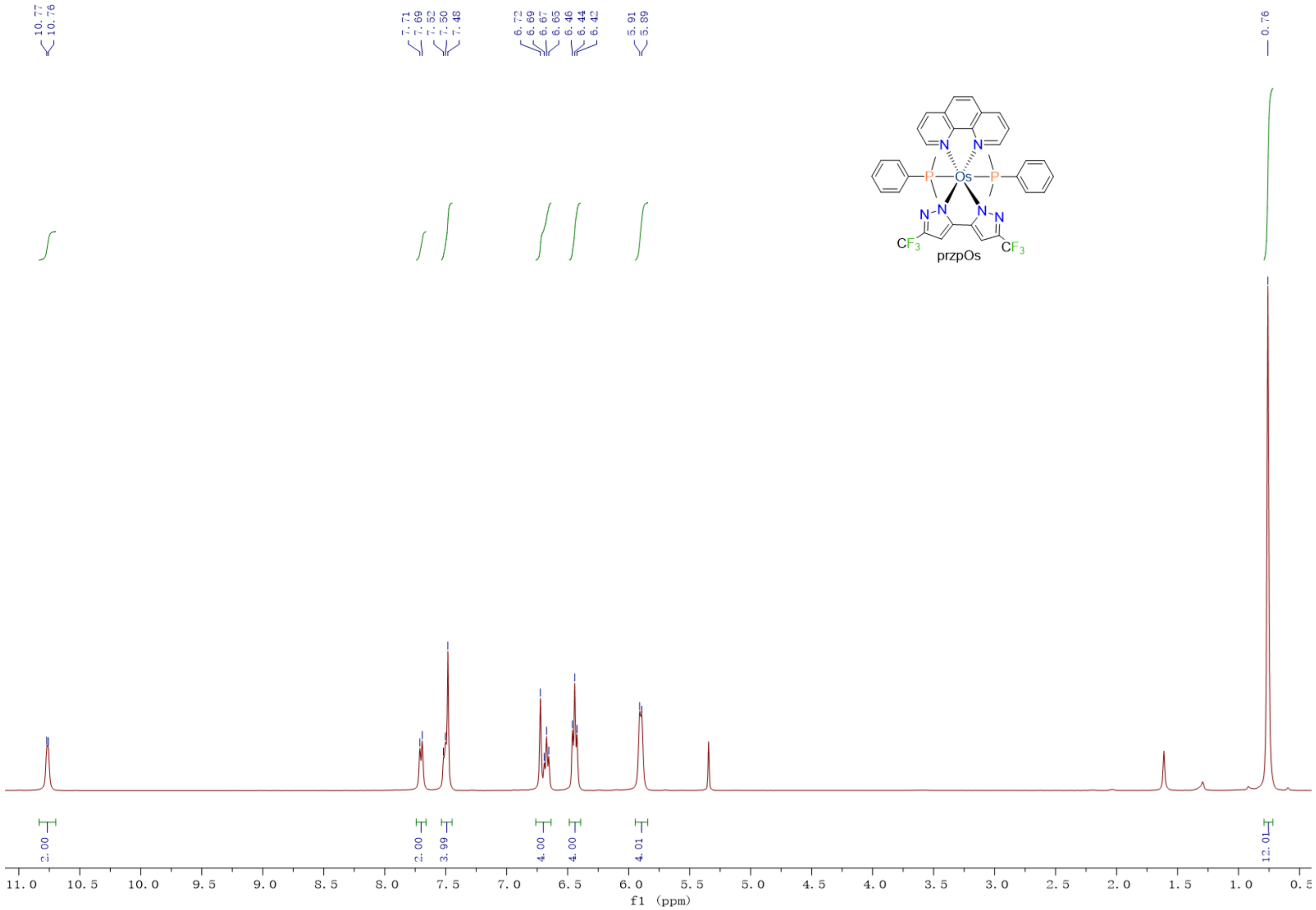

Figure S1. ¹H-NMR of przpOs in CD_2Cl_2. δ 10.76 (d, *J* = 5.4 Hz, 2H), 7.70 (d, *J* = 7.9 Hz, 2H), 7.49 (m, 4H), 6.72 (s, 2H), 6.67 (t, *J* = 7.5 Hz, 2H), 6.44 (t, *J* = 7.6 Hz, 4H), 5.90 (d, *J* = 7.3 Hz, 4H), 0.76 (s, 12H).

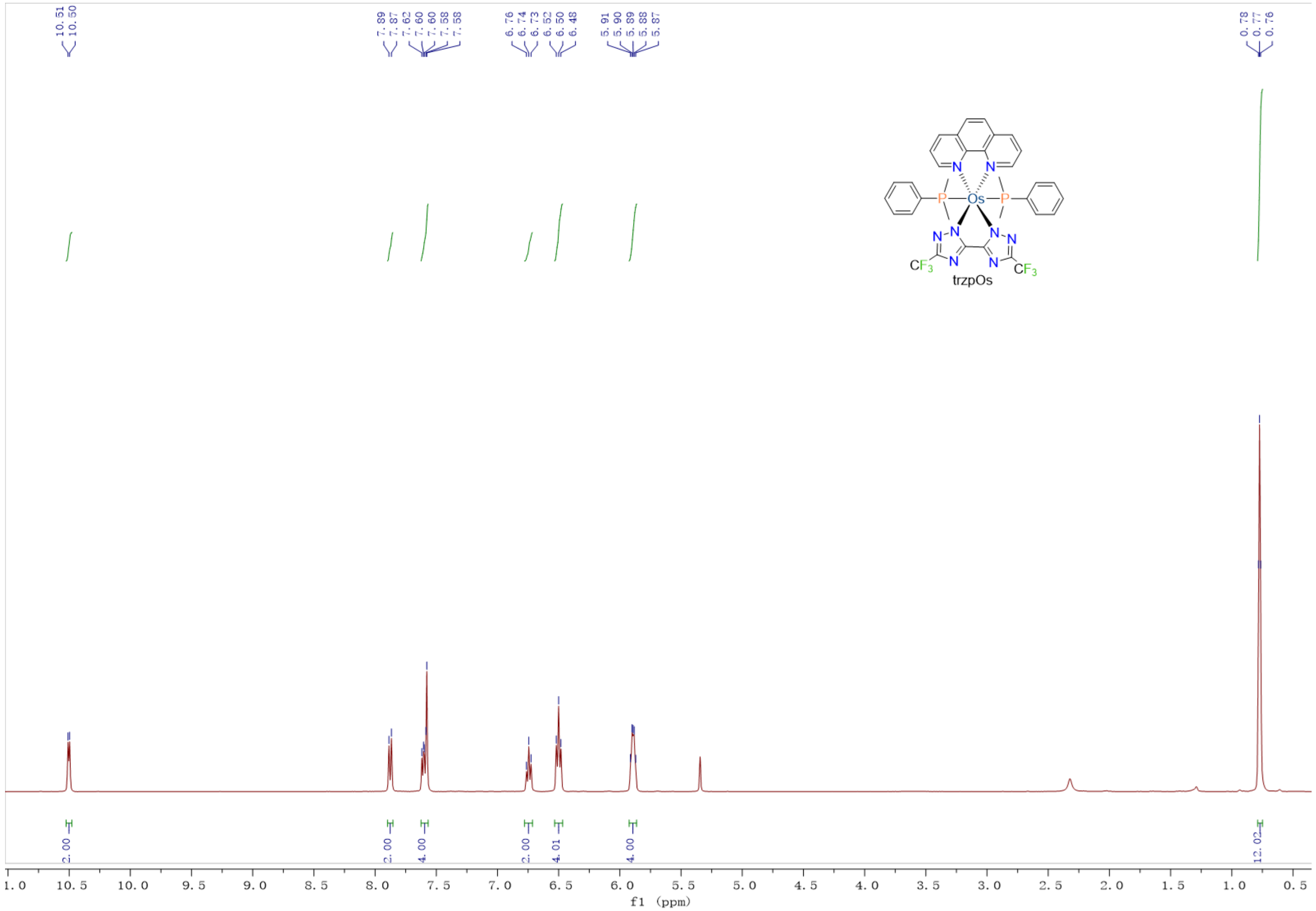

Figure S2. ^{1}H-NMR of trzpOs in CD_2Cl_2. δ 10.50 (d, *J* = 5.3 Hz, 2H), 7.88 (d, *J* = 8.0 Hz, 2H), 7.62-7.57 (m, 4H), 6.74 (t, *J* = 7.5 Hz, 2H), 6.50 (t, *J* = 7.6 Hz, 4H), 5.89 (dt, *J* = 8.4, 4.8 Hz, 4H), 0.77 (t, *J* = 3.4 Hz, 12H).

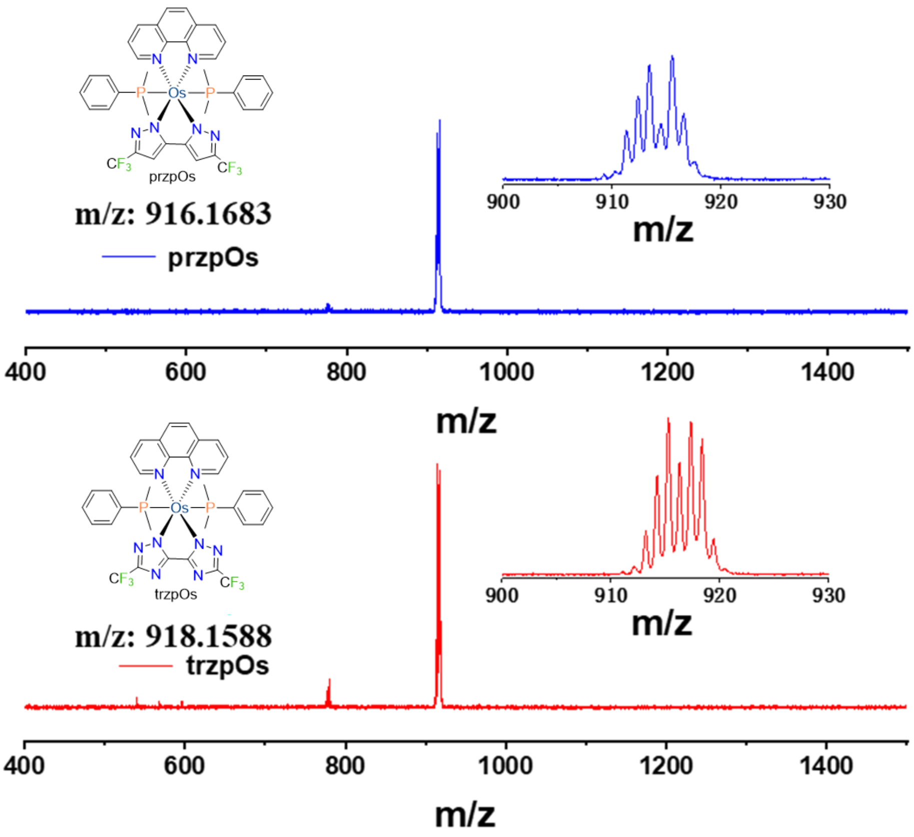

Figure S3. Mass spectroscopy. High-resolution MS of przpOs and trzpOs.

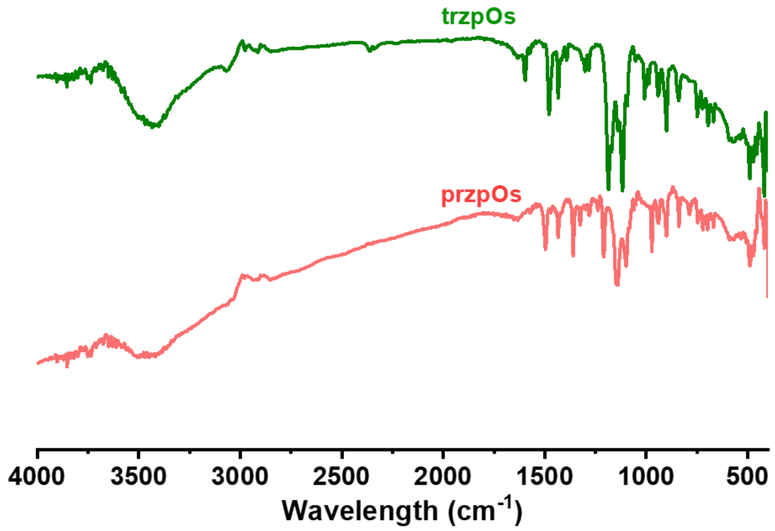

Figure S4. FTIR spectroscopy. FTIR of przpOs and trzpOs.

S1. Single crystal structure and checkCIF/PLATON report of przpOs

Datablock: przpOs

Bond precision: C-C = 0.0084 A; Wavelength=1.54178

Cell: a=36.3907(9) b=10.9407(3) c=19.6531(5)

alpha=90 beta=115.270(1) gamma=90

Temperature: 213 K

	Calculated	Reported
Volume	7075.9(3)	7075.9(3)
Space group	C 2/c	C 2/c
Hall group	-C 2yc	-C 2yc
Moiety formula	$C_{36}H_{32}F_6N_6OsP_2$	
Sum formula	$C_{36}H_{32}F_6N_6OsP_2$	$C_{36}H_{32}F_6N_6OsP_2$
Mr	914.85	914.81
Dx,g cm-3	1.718	1.717
Z	8	8
Mu (mm-1)	8.258	8.258
F000	3600.0	3600.0
F000’	3574.70	
h,k,lmax	45,13,24	45,13,24
Nref	7245	7110
Tmin,Tmax	0.170,0.921	0.606,0.754
Tmin’	0.037	

Correction method= # Reported T Limits: Tmin=0.606 Tmax=0.754

AbsCorr = MULTI-SCAN

Data completeness= 0.981

Theta(max)= 74.444

R(reflections)= 0.0349(5791)

wR2(reflections)= 0.1088(7110)

S = 1.021

Npar= 464

Alert level C

PLAT213_ALERT_2_C Atom F4 has ADP max/min Ratio ..... 4.0 prolat

PLAT213_ALERT_2_C Atom F6 has ADP max/min Ratio ..... 3.5 prolat

PLAT230_ALERT_2_C Hirshfeld Test Diff for N4 --C4 . 6.0 s.u.

PLAT342_ALERT_3_C Low Bond Precision on C-C Bonds .... 0.00835 Ang.

PLAT906_ALERT_3_C Large K Value in the Analysis of Variance ...... 2.270 Check

PLAT911_ALERT_3_C Missing FCF Refl Between Thmin&STh/L=0.600 69 Report

PLAT924_ALERT_1_C The Reported and Calculated Rho(min) Differ by. 1.30 eA-3

PLAT925_ALERT_1_C The Reported and Calculated Rho(max) Differ by. 1.18 eA-3

PLAT971_ALERT_2_C Check Calcd Resid. Dens. 1.07A From Os1 1.56 eA-3

Alert level G

PLAT128_ALERT_4_G Alternate Setting for Input Space Group C2/c I2/a Note

PLAT242_ALERT_2_G Low 'MainMol' Ueq as Compared to Neighbors of C_7 Check

PLAT242_ALERT_2_G Low 'MainMol' Ueq as Compared to Neighbors of C_8 Check

PLAT883_ALERT_1_G No Info/Value for _atom_sites_solution_primary. Please Do !

PLAT912_ALERT_4_G Missing # of FCF Reflections Above STh/L= 0.600 67 Note

PLAT941_ALERT_3_G Average HKL Measurement Multiplicity .... 4.3 Low

PLAT978_ALERT_2_G Number C-C Bonds with Positive Residual Density. 0 Info

PLAT992_ALERT_5_G Repd & Actual _reflns_number_gt Values Differ by 1 Check

0 **ALERT level A** = Most likely a serious problem - resolve or explain

0 **ALERT level B** = A potentially serious problem, consider carefully

9 **ALERT level C** = Check. Ensure it is not caused by an omission or oversight

8 **ALERT level G** = General information/check it is not something unexpected

3 ALERT type 1 CIF construction/syntax error, inconsistent or missing data

7 ALERT type 2 Indicator that the structure model may be wrong or deficient

4 ALERT type 3 Indicator that the structure quality may be low

2 ALERT type 4 Improvement, methodology, query or suggestion

1 ALERT type 5 Informative message, check

Table S1. Photophysical parameters of przpOs and trzpOs.

[Os]	λ_{abs} (nm)	$E_{g,op}$* (eV)	λ_{PL} (nm)	Φ_{PL} (%)	E_{HOMO}/E_{LUMO}** (eV)	$E_{g,H-L}$** (eV)
przpOs	485	2.30	839	0.2	-4.67/-2.16	2.51
trzpOs	456	2.45	783	2.5	-4.99/-2.39	2.60

* $E_{g,op}$ optical band gaps calculated from the edge of MLCT absorption.

**$E_{g,HL}$ HOMO and LUMO levels from DFT calculation;

Table S2. Fitting PL lifetimes of 10^{-5} M przpOs and trzpOs in N_2 and statured CO_2 electrolyte (0.1 M $NaHCO_3$, pH = 8.31).

Samples	τ_1 (ns)	A_1 (%)	τ_1 (ns)	A_2 (%)	τ^* (ns)	k_{et}** (s^{-1})
przpOs	103.0	29.07	1483.6	70.93	1445.4	--
przpOs+CO_2	238.1	40.93	973.0	59.07	866.4	4.62×10^5
trzpOs	86.4	20.56	1624.9	79.44	1604.0	--
trzpOs+CO_2	140.6	38.96	884.3	61.04	815.8	6.02×10^5

*Average lifetime $\tau = (A_1 \times \tau_1^2 + A_2 \times \tau_2^2) / (A_1 \times \tau_1 + A_2 \times \tau_2)$

**k_{et}: electron transfer constant from the excited state of [Os] complex to CO_2

Table S3. Electrochemical CO_2R activities of przpOs and trzpOs.

[Os]	E_1/E_2 (V)	$E_{g,redox}$ (eV)	i_0 (μA)	i_c (μA)	$i_{c\text{-H}}$ (μA)	k_{cat} (s^{-1})	k_{cat}-H (s^{-1})
przpOs	0.64/-1.44	2.08	2.7	7.9	18.1	0.104	0.545
trzpOs	0.82/-1.44	2.26	2.3	7.3	52.9	0.122	6.41

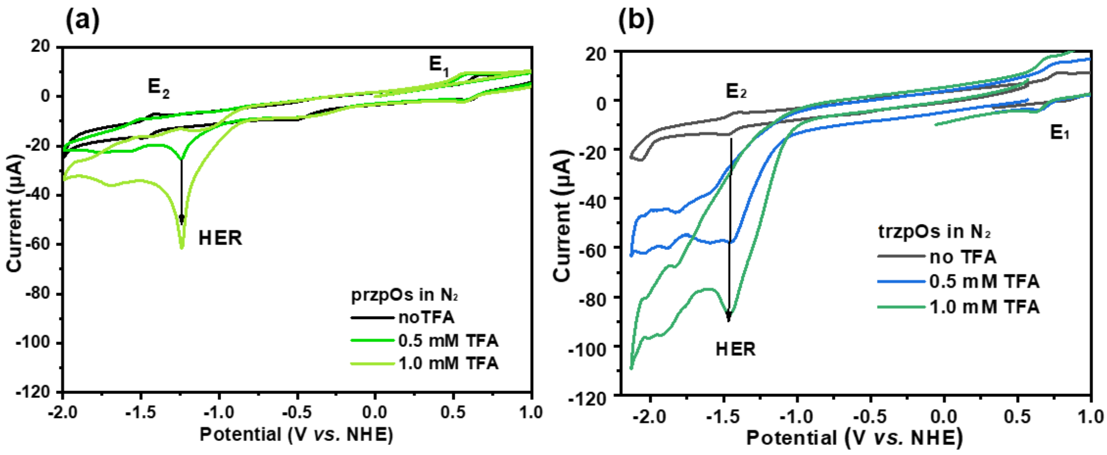

Figure S5. **Electrochemical HER of [Os] complex.** CV curves of 1.0 mM of przpOs (a) and trzpOs (b) in N_2 atmosphere with the addition of proton source of TFA. Without iR corrections.

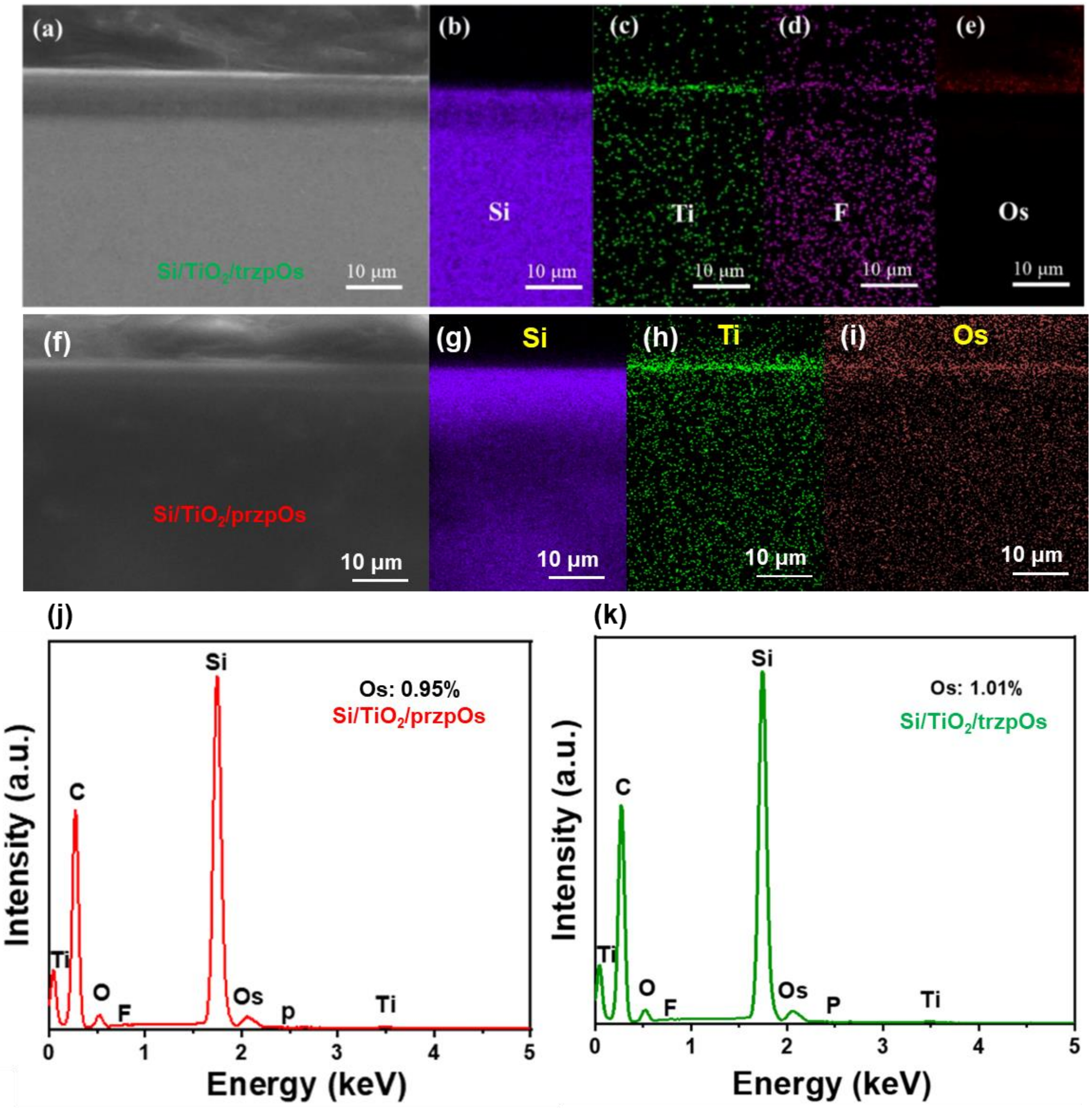

Figure S6. **Element distribution diagram of the electrodes.** (a) Cross-sectional SEM image of Si/TiO_2/trzpOs, and the related elemental mapping of Si (b), Ti (c), F (d) and Os (e). (f) Cross-sectional SEM image of Si/TiO_2/przpOs, and the related elemental mapping of Si (g), Ti (h) and Os (i). EDX spectra of Si/TiO_2/przpOs (j) and p-Si/TiO_2/trzpOs (k).

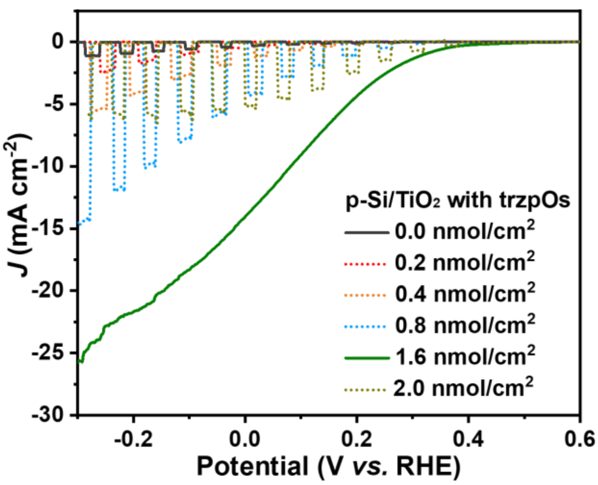

Figure S7. Optimization of catalyst dosage. LSV curves of Si/TiO_2/trzpOs electrode with different amounts of trzpOs in the CO_2-saturated 0.1 M $KHCO_3$ electrolyte under chopped illumination of AM 1.5G, and without iR corrections.

Table S4. PEC CO_2R of Si/TiO_2 with and without [Os] complexes catalyst.

Samples	j_{ph}@0V_{RHE} (mA/cm^2)	E_{on} (V_{RHE})	FE_{H2} (%)	FE_{CO} (%)	FE_{CH4} (%)
Si/TiO_2	-0.34 (N_2)<br>-0.39 (CO_2)	0.24	85.2±2.0	4.7±2.0	0
Si/TiO_2/przpOs	-4.11 (N_2)<br>-8.43 (CO_2)	0.56	8.6±3.1	1.5±1.3	89.8±3.0
Si/TiO_2/trzpOs	-4.38 (N_2)<br>-14.11 (CO_2)	0.52	6.5±3.5	1.7±1.8	91.8±3.1

Table S5. Si-based photocathodes for PEC CO_2R.

Photocathode	CO_2R catalyst	Reaction conditions	Performance	Ref.
B-doped p-Si	Fe porphyrin complex	MeCN/5% DMF (v/v), 0.1 M $TBABF_4$, -1.11 V_{SCE}, 90 mW cm^{-2} 650 nm illumination, 6 h	FE of over 80% for CO evolution	1
p-Si nanowire arrays	Sn NPs	0.1 M $KHCO_3$, -0.85 V_{RHE}, AM 1.5G, 3 h	FE of 40% for HCOOH evolution	2
pn^+-Si	RA–Au film	0.2 M $KHCO_3$, −0.03 V_{RHE}, simulated 1 sun illumination,<br>5-60 min	FE of 91% for CO evolution	3
p-Si photocathode	Particulate Ag film	0.5 M $KHCO_3$, -0.5 V_{RHE}, 0.5 sun illumination, 1 h	FE of over 90% for CO evolution	4
TiO_2-protected n^+p-Si nanowire	Au_3Cu NPs	0.1 M $KHCO_3$, -0.2 V_{RHE}, 20 mW cm^{-2} 740 nm illumination, 0.5 h	FE of 76.6% for CO evolution	5
ZnO/GaN/n^+p-Si	Cu NPs	0.5 M $KHCO_3$, -0.33 V_{RHE}, 300 W Xe lamp 800 mW cm^{-2}, 100 min	FE of 20% for CO evolution	6
p-Si/TiO_2	CotpyP	0.1 M $TBABF_4$ in 7:3 MeCN:H_2O, -1.0 V vs Fc^+/Fc, AM1.5G, > 400 nm, 8 h	FE of 53% for CO evolution	7
p-Si/TiO_2	CoPcP complex	0.5 M $KHCO_3$, -0.53 V_{SHE}, AM1.5G illumination (>400 nm), 120 min	FE of 66% for CO evolution	8
p-Si/TiO_2	przpOs complex	0.1 M $KHCO_3$, 0 V_{RHE}, AM1.5G illumination, 100 min	FE of 89.8±3.0% for CH_4	**This work**
	trzpOs complex		FE of 91.8±3.1% for CH_4	

$TBABF_4$: tetrabutylammonium tetrafluoroborate.

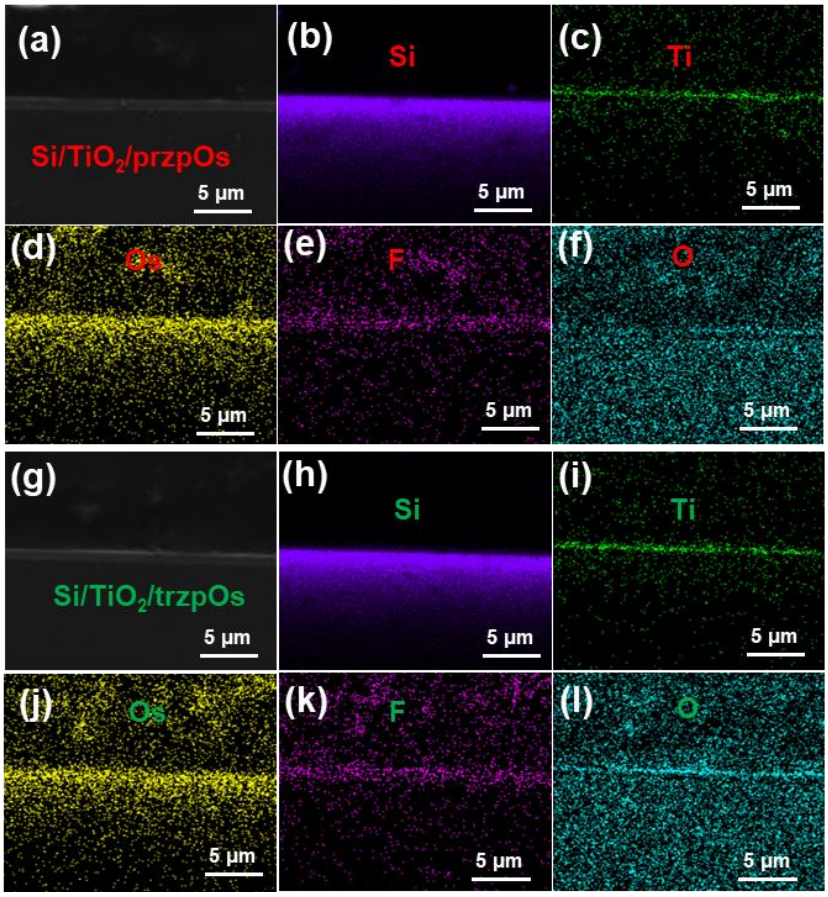

Figure S8. The element distribution of electrodes after PEC test. (a) Cross-sectional SEM images of Si/TiO_2/przpOs after PEC test, and the related elemental mapping of Si (b), Ti (c), Os (d), F (e) and Os (f). (g) Cross-sectional SEM images of Si/TiO_2/trzpOs after PEC test, and the related elemental mapping of Si (h), Ti (i), Os (j), F (k) and Os (l).

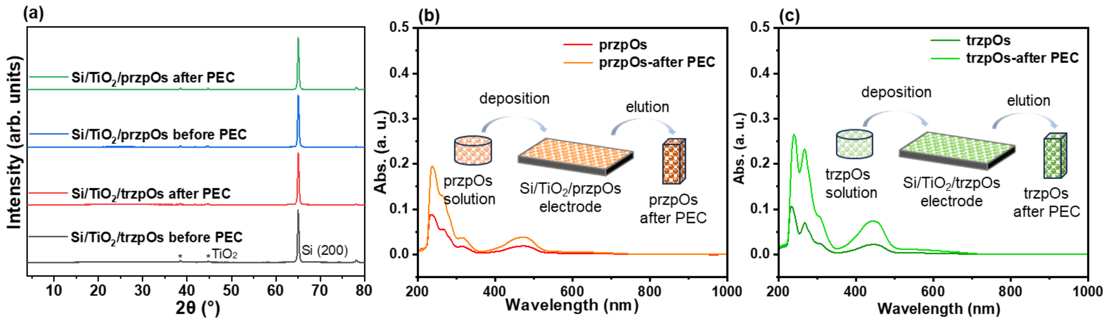

Figure S9. Stability of electrodes and catalysts. (a) XRD pattern of Si/TiO_2/przpOs and Si/TiO_2/trzpOs electrodes before and after PEC tests. (b) UV-vis absorption spectra of przpOs solution and after PEC test. (c) UV-vis absorption spectra of trzpOs solution and after PEC tests. Inset figures show the preparation process of the [Os] sample after the elution from the electrode after PEC tests.

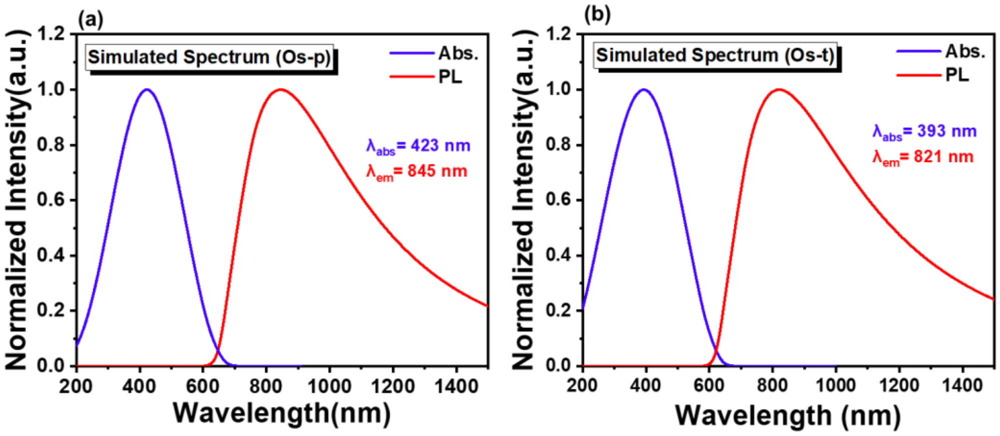

Figure S10. Simulated absorption and luminescence spectra. DFT calculation of absorption and luminescence spectra of przpOs (a) and trzpOs (b).

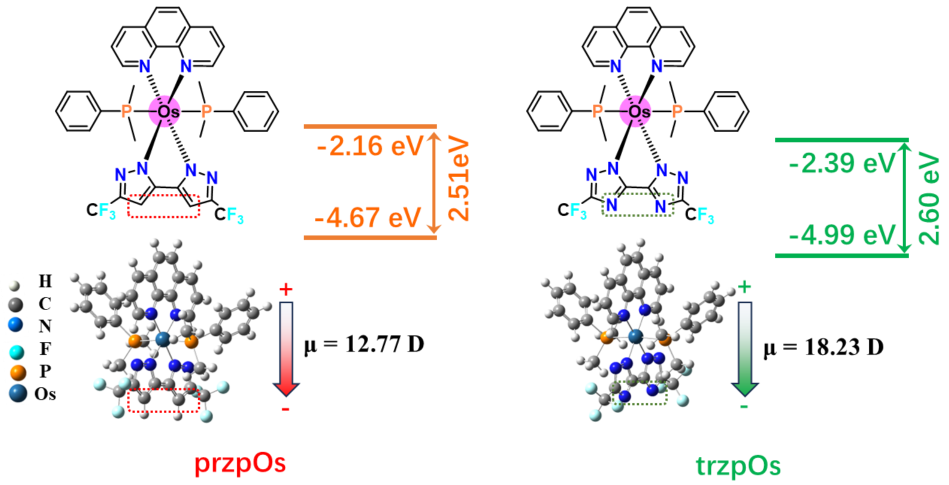

Figure S11. Optimized model structure and energy levels. The calculated molecular geometries, HOMO, LUMO energy levals, $E_{g,H\text{-}L}$ band gaps and dipole moments of przpOs and trzpOs.

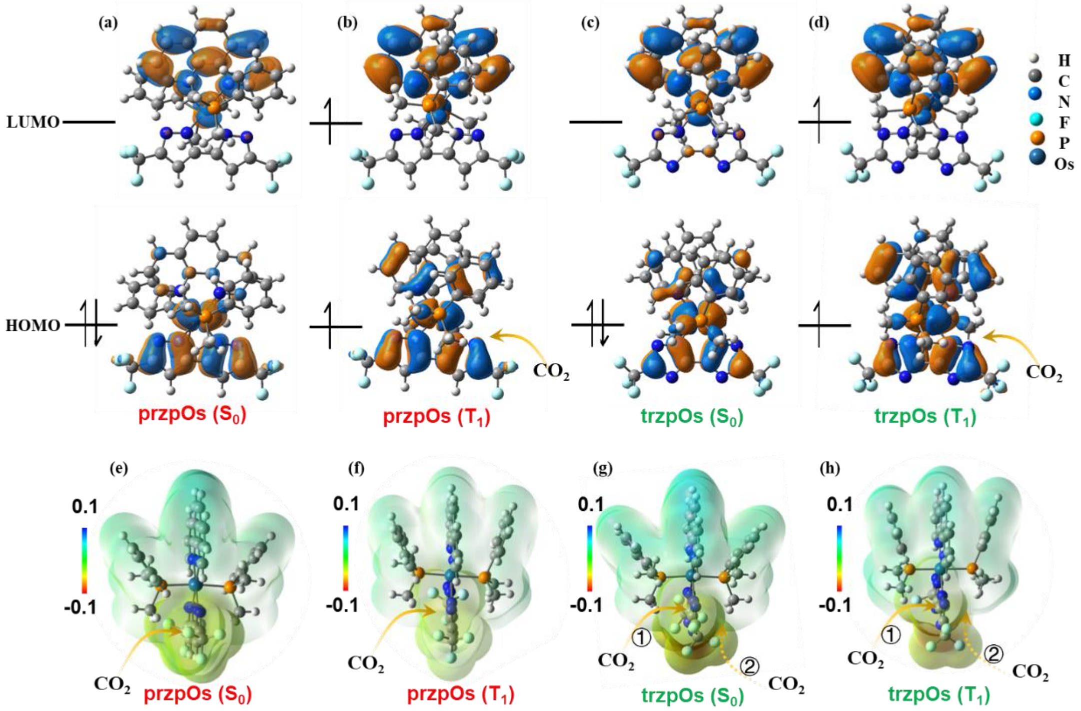

Figure S12. The orbital distribution and electrostatic potential of singlet and triplet states. The distributions of HOMO and LUMO of przpOs in S_0 (a) and T_1 (b) states, and trzpOs in S_0 (c) and T_1 (d) states. Electrostatic potential of przpOs in S_0 (e) and T_1 (f) states, and trzpOs in S_0 (g) and T_1 (h) states, respectively.

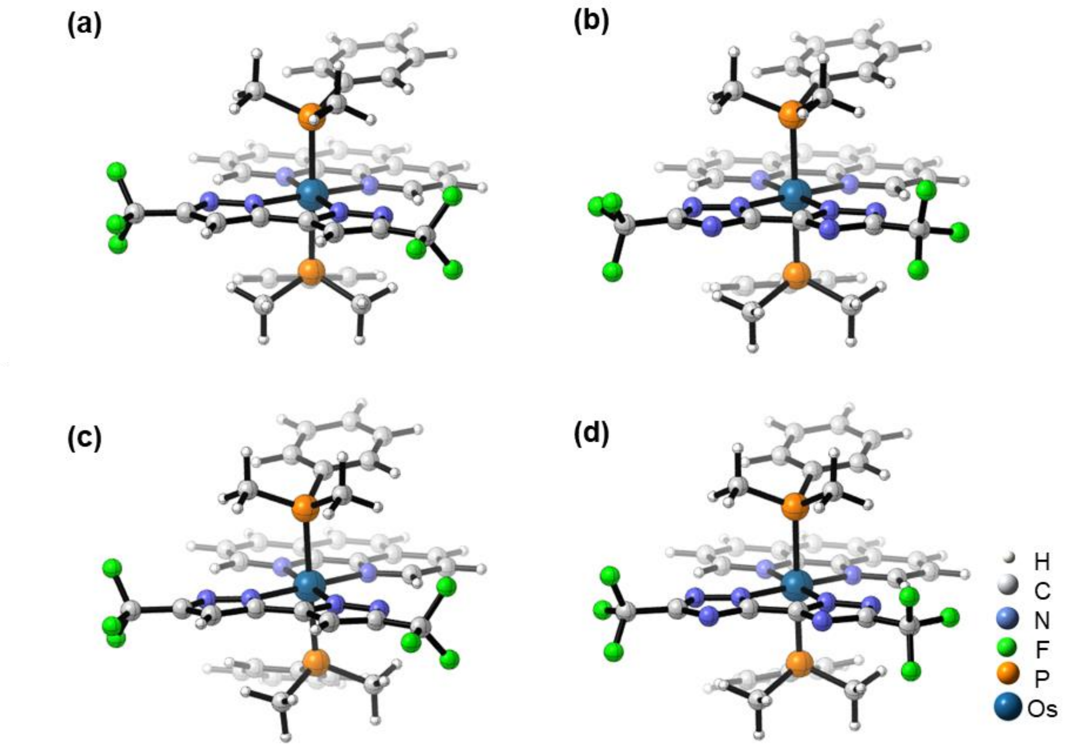

Figure S13. Calculated przpOs and trzpOs in S_0 and T_1 states. S_0 state of przpOs (a) and trzpOs (b), and T_1 state of przpOs (c) and trzpOs (d), respectively.

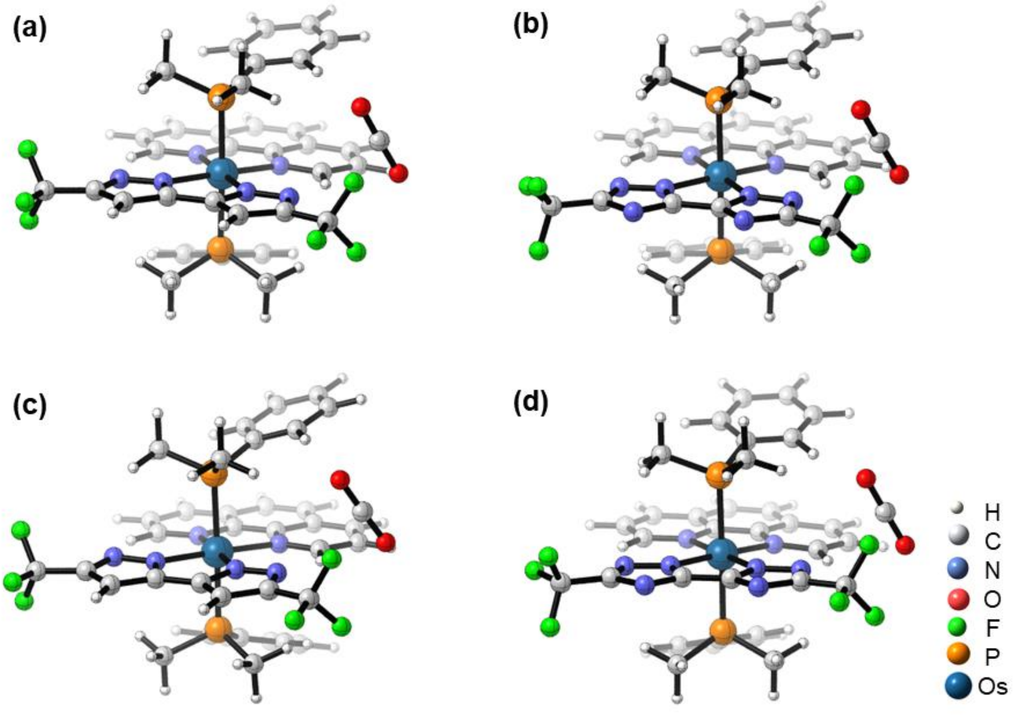

Figure S14. Calculated *CO_2 intermediates in S_0 and T_1 states. Adsorbed *CO_2 in S_0 state of przpOs (a) and trzpOs (b), and T_1 state of przpOs (c) and trzpOs (d), respectively.

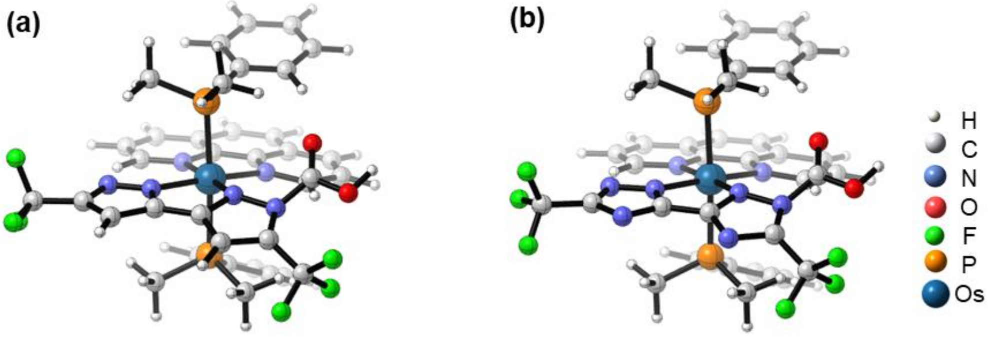

Figure S15. Calculated configurations of *COOH intermediate. przpOs (a) and trzpOs (b).

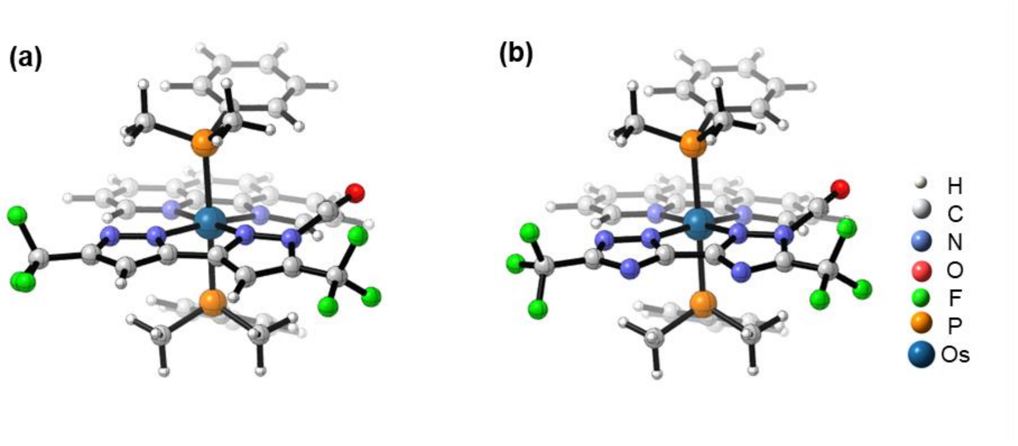

Figure S16. Calculated *CO intermediate. przpOs (a) and trzpOs (b).

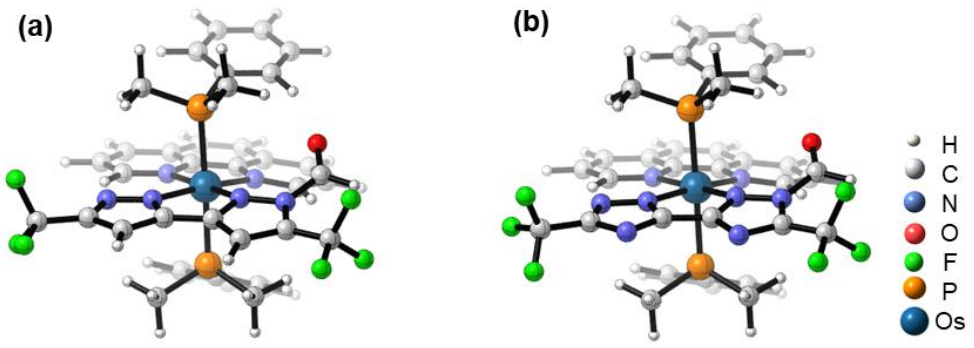

Figure S17. Calculated *CHO intermediate. przpOs (a) and trzpOs (b).

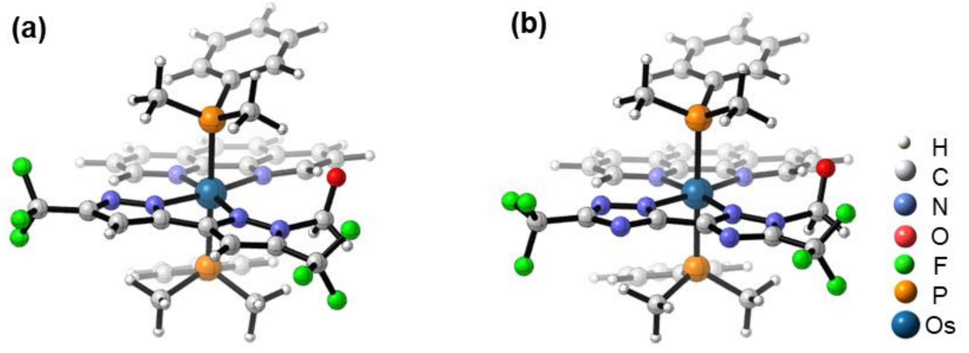

Figure S18. Calculated *CH_2O intermediate. przpOs (a) and trzpOs (b).

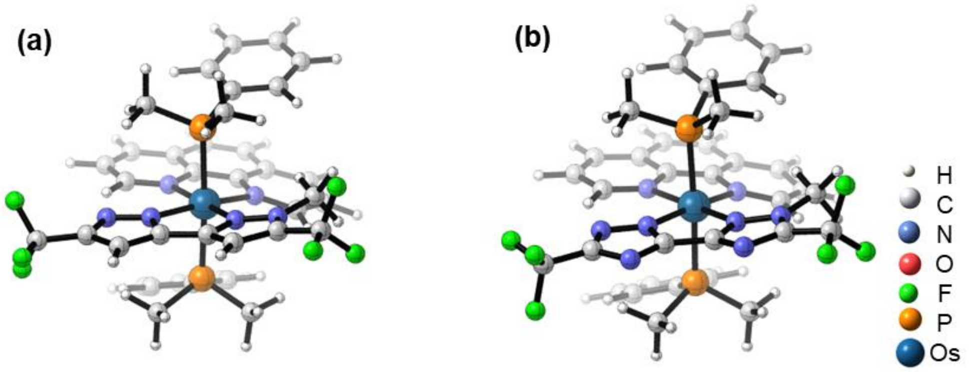

Figure S19. Calculated *CH_3 intermediate. przpOs (a) and trzpOs (b).

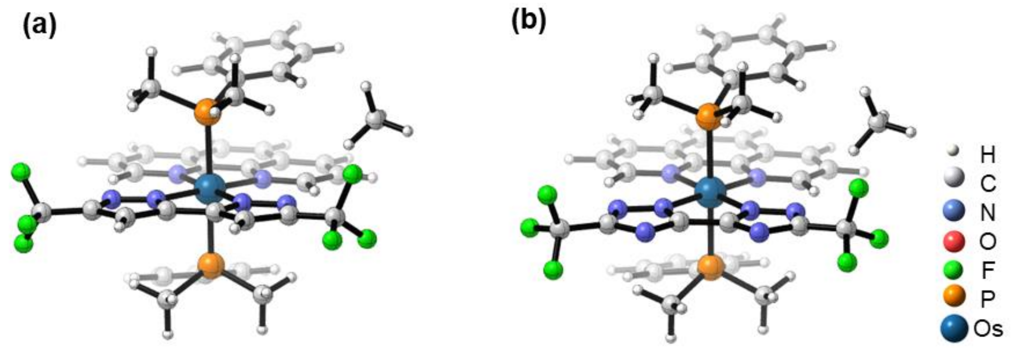

Figure S20. Calculated *CH_4 intermediate. przpOs (a) and trzpOs (b).

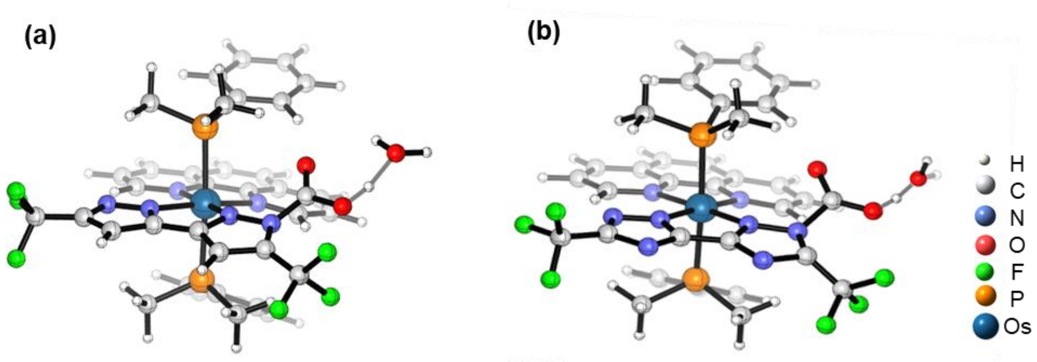

Figure S21. Calculated intermediates for of direct protonation. przpOs (a) and trzpOs (b).

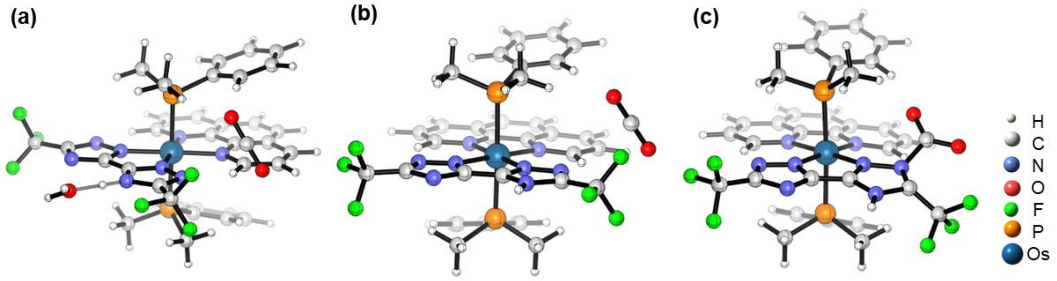

Figure S22. Calculated protonation intermediates *via* nitrogen heteroatom ferrying. Protonation of nitrogen heteroatom (a), intermediate of NH-*CO_2 (b), and proton transfer from NH to *CO_2 (c).

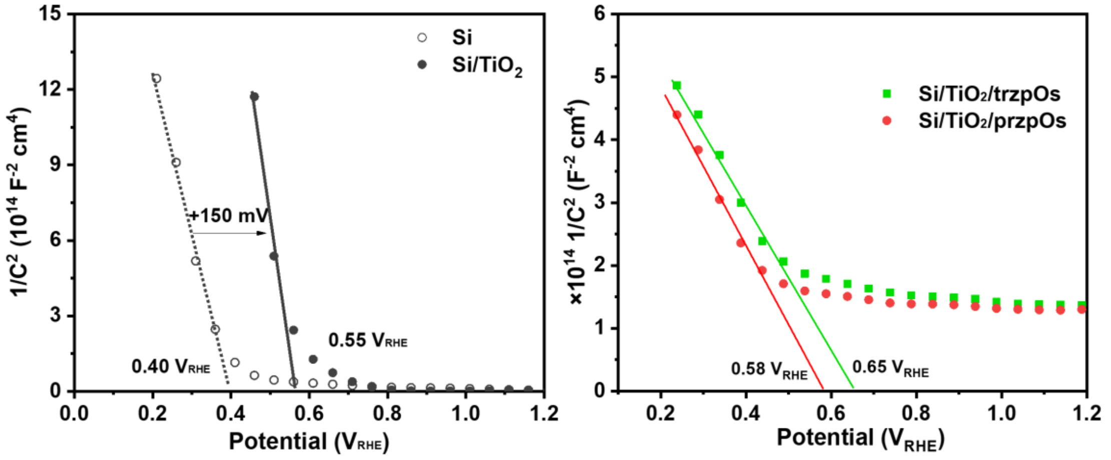

Figure S23. Mott-Schottky plots. Mott-Schottky of black Si, Si/TiO_2, Si/TiO_2/przpOs and Si/TiO_2/trzpOs at a frequency of 1000 Hz.

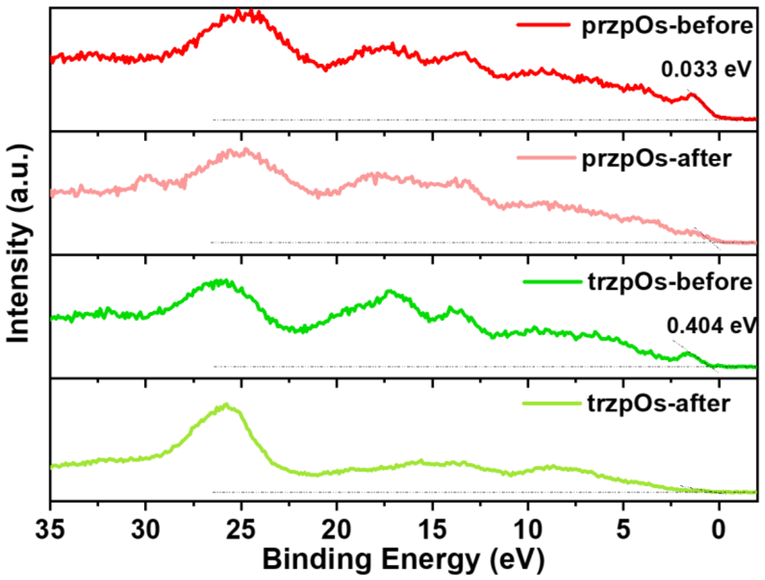

Figure S24. Valence bands of Si/TiO_2/[Os] electrodes. XPS valence band spectra Si/TiO_2/przpOs and Si/TiO_2/trzpOs before and after PEC measurements. The first fitting lines show the linear extrapolation of the curves for deriving the valance band edge position, that is, E_{VB}-E_F value.

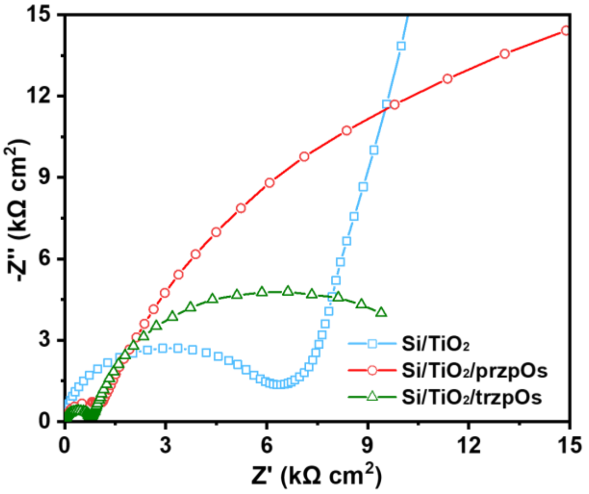

Figure S25. Electrochemical impedance analysis. Nyquist plots of Si/TiO_2, Si/TiO_2/przpOs and Si/TiO_2/trzpOs in CO_2-saturated 0.5 M Na_2SO_4 in light.

Table S6. EIS fitting data of p-Si/TiO_2 electrodes with and without [Os] catalyst

Samples	R_s (Ω cm^2)	$R_{ct,1}$ (Ω cm^2)	CPE_{sc}-T	CPE_{sc}-P	$R_{ct,trap}$ (Ω cm^2)	CPE_2-T	CPE_2-P
Si/TiO_2	117.0	14909	1.460E-10	1.00	561760	3.523E-7	0.72
Si/TiO_2/ przpOs	32.38	9478	1.253E-8	0.80	1.036E6	2.385E-7	0.84
Si/TiO_2/ trzpOs	21.44	8292	2.309E-9	0.97	155040	1.105E-6	0.80

The EIS data are fitted using the equivalent circuits shown following:

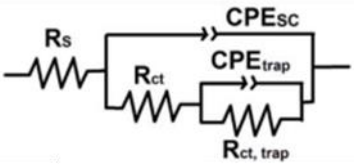

The constant phase elements (CPE) are used instead of the standard capacitance (C) in equivalent circuits due to the non-ideal capacitive behavior. $C = CPE(\omega_{max})^{n/1}$, where ω_{max} is the frequency at which the imaginary value of the impedance has the maximum, and n is the empirical constant that describes the electrical behavior of CPE (CPE-T).

Reference:

1 Alenezi, K., Ibrahim, S. K., Li, P. & Pickett, C. J. Solar Fuels: Photoelectrosynthesis of CO from CO_2 at p-Type Si using Fe Porphyrin Electrocatalysts. *Chem. Eur. J.* **19**, 13522-13527 (2013).

2 Choi, S. K. *et al.* Sn-Coupled p-Si Nanowire Arrays for Solar Formate Production from CO_2. *Adv. Energy Mater.* **4**, 1301614 (2014).

3 Song, J. T. *et al.* Nanoporous Au Thin Films on Si Photoelectrodes for Selective and Efficient Photoelectrochemical CO_2 Reduction. *Adv. Energy Mater.* **7**, 1601103 (2017).

4 Hu, Y. *et al.* Designing effective Si/Ag interface via controlled chemical etching for photoelectrochemical CO_2 reduction. *J. Mater. Chem. A* **6**, 21906-21912 (2018).

5 Kong, Q. *et al.* Directed Assembly of Nanoparticle Catalysts on Nanowire Photoelectrodes for Photoelectrochemical CO_2 Reduction. *Nano Lett.* **16**, 5675-5680 (2016).

6 Chu, S. *et al.* Tunable Syngas Production from CO_2 and H_2O in an Aqueous Photoelectrochemical Cell. *Angew. Chem. Int. Ed.* **55**, 14262-14266 (2016).

7 Leung, J. J. *et al.* Solar-driven reduction of aqueous CO_2 with a cobalt bis(terpyridine)-based photocathode. *Nat. Catal.* **2**, 354-365 (2019).

8 Roy, S. *et al.* Electrocatalytic and Solar-Driven Reduction of Aqueous CO_2 with Molecular Cobalt Phthalocyanine-Metal Oxide Hybrid Materials. *ACS Catal.* **11**, 1868-1876 (2021).
